# The impact of career calling on higher vocational nursing students’ learning engagement: The mediating roles of career adaptability and career commitment

**DOI:** 10.3389/fpsyg.2023.1111842

**Published:** 2023-03-22

**Authors:** Jingyuan Chen, Xiaoxia Zhang

**Affiliations:** ^1^Information Technology Office, Zhengzhou Railway Vocational & Technical College, Zhengzhou, China; ^2^Department of Pharmacy, Zhengzhou Railway Vocational and Technical College, Zhengzhou, China

**Keywords:** career calling, learning engagement, career adaptability, career commitment, higher vocational nursing students

## Abstract

Learning engagement is an important predictor of academic success and a key indicator of educational quality. It has therefore attracted considerable research interest, with previous studies exploring how to enhance engagement *via* pedagogical approaches, strategies, and content, as well as through teacher-student interaction. However, the relationship between individual learners’ internal mechanisms and learning engagement has yet to be investigated in depth. Accordingly, the present study explored the direct effects of career calling on higher vocational nursing students’ learning engagement and the mediating roles of career adaptability and career commitment *via* a parallel mediation model. Data were collected from 388 nursing students at two higher vocational colleges in China and the hypotheses were tested using correlation and regression analyses. The results showed that career calling imparted a significant positive effect on the nursing students’ engagement in learning, with career adaptability and career commitment mediating this relationship. These findings highlight the importance of promoting learning engagement among nursing students and the need to carefully design instructional activities for the healthcare profession.

## 1. Introduction

*Learning engagement* denotes a state of sustained, positive affect toward learning characterized by vigor, dedication, and absorption (Schaufeli et al., 2002). An important predictor of academic success and indicator of educational quality, learning engagement has recently attracted considerable research attention. It is positively related to learners’ academic achievement and experience of attendance as well as deep learning, reduced dropout rates, increased learner satisfaction with school, future career development levels, wellbeing, and critical thinking skills (Howell, 2009; Trenholm et al., 2019; Jian et al., 2022). However, in China as in other contexts, insufficient engagement in learning among university students is a widespread phenomenon. The problem is particularly prominent among higher vocational nursing students: under powerful pressures to learn, such learners exhibit a lack of interest, initiative, motivation, and identification with their profession, a lack of goal-setting, and burnout; they may also have insufficient access to high-quality learning opportunities (Salam et al., 2013; He et al., 2018). As nurses of the future, these students’ lack of engagement not only affects their learning and future levels of professional competence but also determines the quality of nursing available to all users of the country’s healthcare services. It is therefore crucial to explore the antecedents of poor learning engagement among nursing students since doing so will improve the quality of their learning and develop their professional competence.

*Career calling* has been described as “the practice of a particular life role in a transcendent calling that originates in and transcends the self, i.e., in a way that demonstrates or gains a sense of purpose or meaning and other-oriented values and goals as a fundamental source of motivation” (Dik and Duffy, 2009). The concept emphasizes people’s sense of ulteriority that aligns them with societal obligations, familial heritage, or religious convictions. This sense makes individuals feel that their work—either directly or indirectly—helps others or society as a whole and thus allows them to experience a deep sense of meaning and purpose in life. Career calling has attracted much interest from practitioners working in management and education as well as in positive, occupational, industrial, and organizational branches of psychology. Numerous studies show that career calling is positively related to wellbeing outcomes such as work engagement, life happiness, and job satisfaction; it is particularly important for jobs that are challenging and require extra time, energy, and effort. For example, although nurses in developing countries face problems such as low pay, overload, poor working environments, and the possibility of occupational exposure to harmful substances, those with higher levels of career calling are more meticulous and conscientious, make fewer errors, and report fewer turnover intentions (Afsar et al., 2019). Similarly, while the doctors in Bott et al.’s research Bott et al. (2017) generally found their work stressful and challenging, those with stronger career calling also tended to report more positive work experiences. They reported feeling more motivated, productive, and responsible for using their skills to help others. Career calling also exists among university students and is positively related to their career-related decision-making behavior (Hunter et al., 2010). Students with stronger career calling tend to enjoy greater access to educational resources (Duffy and Sedlacek, 2010). They are also more proactive in their pursuit of personal growth and achieve higher levels of academic satisfaction (Duffy et al., 2011a).

Socio-cognitive theories of career development have been used to explore the links between career calling, self-efficacy, expectations of career outcomes, and engagement in learning (Chen et al., 2016; Shang et al., 2022). However, this research has tended to focus on single samples of pre-service teachers rather than students of other professions. In the current study, we wished to deepen understanding of the unique relationship between career calling and learning engagement among nursing students. Such uniqueness exists in these students’ orientations to professional learning, the complexity of course content, the considerable academic pressures they face, and their particular need for a sense of career calling compared to students on other professional courses. Accordingly, the present study explored the factors influencing the learning engagement of nursing students to enable more effective evaluation and reform of their current professional development programs. Our study also aimed to help improve employment outcomes in a general sense, given that the career adaptability and commitment of students in all fields is linked to their engagement in university learning. Students in all fields prepare for their future careers by learning professional theoretical knowledge and practicing vocational skills at university, and their career adaptability and commitment also influence their learning engagement. In summary, this study investigated the relationship between career calling and learning engagement, and the mediating role of career adaptability and career commitment among nursing students.

## 2. Theoretical background and study hypotheses

### 2.1. Career calling and learning engagement

A professional calling can be defined as the sustained, meaningful passion for a particular domain that infuses one’s choice of career with a broader sense of meaning, purpose, and direction (Dobrow and Tosti-Kharas, 2011). Relatedly, self-determination theory states that individuals choose their behavior to meet their needs in a particular environment, engaging actively in behaviors or tasks that benefit their development. Several studies have shown that across different work areas, career calling has significant and positive associations with positive work experiences such as job engagement and pleasure, wellbeing, and career commitment while it is negatively associated with less beneficial experiences. For example, Duffy et al.’s (2012) online survey of 201 workers in various industries found that career calling was positively correlated with their job satisfaction. Ke et al. (2020) reported that career calling positively affected the learning behavior of civil servants. Dobrow and Tosti-Kharas (2011) surveyed employees within the fields of music, arts, business, and management, finding that career calling positively impacted their job engagement, job satisfaction, career-related self-efficacy, and clarity of professional identity. Lastly, Zhou et al. (2020) explored the relationship between career calling and work fatigue among Chinese police officers during the COVID-19 pandemic: their results indicated that a moderate level of career calling boosted work-related personal resources and could therefore reduce negative work experiences such as work fatigue and role overload.

Unlike adults in the workplace, university students may perceive career calling as a spiritual motivator. Students often actively prepare themselves for their future professional lives by constructing an ideal future work self *via* career calling, which motivates further engagement in anticipatory or future-oriented career behavior (Strauss et al., 2012). Career calling is therefore closely linked to undergraduate students’ career development. Examining this relationship, Duffy and Sedlacek (2007) found it was positively related to career decidedness, comfort, self-clarity, and work-choice salience, and negatively related to indecisiveness and lack of educational information. Similarly, Hirschi and Herrmann (2013) reported that career calling was significantly and positively related to career planning, decidedness, and self-efficacy among university students while Duffy et al. (2014) found it promoted individual growth initiatives among university students. The present study defines the learning engagement of nursing students as their active, positive, and sustained commitment to comprehensive and up-to-date professional learning, which can be motivated by career calling. In this way, learners can achieve professional self-worth and self-satisfaction. Accordingly, H1 was as follows:

*H1*: Career calling has a positive effect on nursing students’ learning engagement.

### 2.2. The mediating roles of career commitment and career adaptability

*Career commitment* is the intensity of motivation that an individual shows at work to achieve his or her goals (Hall et al., 1971). It indicates the extent to which an individual identifies with his or her occupation by taking responsibility and fulfilling professional obligations. Career commitment positively impacts job skills, reduces turnover rates, and increases job satisfaction (Aryee and Tan, 1992; Chang, 1999) because committed individuals tend to take a more active role in their work and strive to create an environment conducive to their professional development.

Research has repeatedly highlighted the strong relationships between career calling and job-related outcomes such as job engagement and satisfaction. But how are such relationships established? Duffy et al. (2012) argue that career commitment mediates the positive association between career calling and positive job-related outcomes. For example, Duffy et al.’s (2011b) survey of 370 staff at a Western research university found that career commitment fully mediated the relationship between people’s sense of calling and their job satisfaction, meaning that individuals with stronger career calling were more satisfied with their jobs because they identified more with their work. Afsar et al. (2019) found that nurses with a stronger sense of career calling tended to display greater commitment to their careers, which significantly reduced their turnover rate and raised their workplace performance. In addition, career commitment may help motivate professional development and learning: findings from Ke et al.’s (2020) survey of 367 Chinese civil servants showed it mediated the link between their sense of vocation and learning. Therefore, strong career identifications motivated these professionals to maintain self-directed learning behavior. Based on these findings, we hypothesized that nursing students with a strong sense of career calling would display greater learning engagement due to the mediation of their career commitment:

*H2*: Career commitment mediates the relationship between career calling and nursing students’ learning engagement.

Savickas’s (2002) career construction theory proposed that career development results when the individual’s adaptation to the environment is aligned with his or her psychology rather than his or her intrinsic structural maturity. One core concept of career construction theory is *career adaptability*, which refers to the individual’s psychological readiness and resources for current or anticipated tasks related to his or her career. Essentially, career adaptability is a self-regulatory ability that drives individuals to continually adjust their environmental responses. It consists of four dimensions: concern, control, curiosity, and confidence. Concern refers to how people consider their sense of purpose when setting career goals. Control enables individuals to manage and shape their own careers. Curiosity refers to the individual’s exploration of the possibilities for self-fulfillment and opportunities in their careers. Finally, confidence refers to an individual’s sense of efficacy in overcoming potential career obstacles.

Career construction theory predicts that people with high levels of career adaptability will have more mental resources and greater work-related adaptability. They carefully follow future trends and prepare to proactively face challenges, adapt to changes in their career environments, and engage in new forms of work. Career adaptability not only correlates positively and significantly with job performance but also affects students’ academic development. For example, Yu and Li (2015) found that students with stronger career adaptability were highly motivated to learn, actively constructed learning goals, and planned their coursework and self-growth well, producing excellent academic outcomes. Guo et al. (2014) found that career adaptability played a positive role in enhancing the professional competence of undergraduates.

Psychology researchers define career calling as the deepest level of an individual’s perception of subjective professional success. It provides individuals with “metacompetencies” (Hall and Chandler, 2005), helping them to achieve psychological success even when objective success eludes them. One such metacompetency is *career adaptability*, which is the ability of individuals to remotivate themselves and update their job-related knowledge and skills. It also aids self-directed learning and the ability to switch careers. The scant research into career calling and career adaptability suggests the two are significantly correlated. For example, Praskova et al. (2014) uncovered a moderately strong correlation based on survey data from 216 Australian students gathered at two points 6 months apart. Yang and Chen (2020) found that nurses’ career calling was positively correlated with their career adaptability. They also found that career adaptability positively influenced the nurses’ occupational embeddedness. Drawing on the findings summarized above, we proposed the following hypothesis:

*H3*: Career adaptability mediates the relationship between career calling and nursing students’ learning engagement.

**Figure 1:**
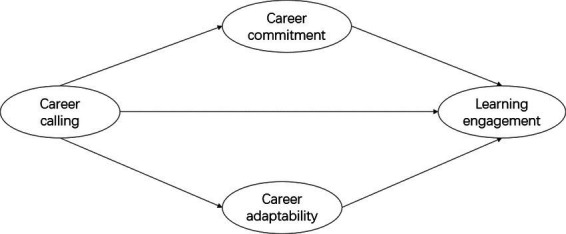
Hypothesized model of mediated relationships between career calling and learning engagement.

In our investigation of the internal mechanism of the mediated effects of career calling on learning investment, hypotheses 1–3 were exploratory, reflecting the fact that research on nursing students’ learning engagement in China is still in its infancy. We hoped to provide a solid theoretical foundation to enhance such students’ investments in learning and to improve the quality of education they receive. Figure 1 displays our theoretical model.

## 3. Materials and methods

### 3.1. Participants and procedure

The online questionnaire (Wen Juanxing) was sent to participants *via* WeChat or email. After providing their informed consent, the respondents completed all the items and submitted their questionnaires. All responses were anonymous and stored securely for access by the study team solely for the purposes of this research. Approval for the study was granted by the Ethics Committee of Zhengzhou Railway Vocational & Technical College.

The participants (*N* = 403) were nursing students from two higher vocational colleges in a central Chinese province. A total of 15 responses were excluded because 80% or more of the answers were the same or the response time was too fast (less than 2 min). This left 388 questionnaires, a return rate of 96.28%. Male students completed 92 (23.71%) of the questionnaires and female students, 296 (76.29%). By grade level, 135 were freshmen (34.79%), 141 were sophomore students (36.34%) and 112 were juniors (28.87%). A summary of respondent characteristics is presented in Table 1.

**Table 1:** Demographic characteristics of respondents.

Variables	N (% of 388)
Gender	
Male	92 (23.71%)
Female	296 (76.29%)
Grade	
Freshmen (year 1)	135 (34.79%)
Sophomores (year 2)	141 (36.34%)
Juniors (year 3)	112 (28.87%)
Home location	
Rural towns	320 (82.5%)
Small and medium-sized cities	52 (13.4%)
Big cities	16 (4.1%)
Categories of high school	
Secondary vocational and technical college	48 (12.4%)
Ordinary high school	304 (78.4%)
Key high school	36 (9.3%)
Type of college application	
Nursing as own choice	216 (55.7%)
Nursing not own choice	172 (44.3%)
The highest level of education of the participant’s father	
Primary school and below	116 (29.9%)
Secondary or technical secondary school	208 (53.6%)
Higher vocational School	52 (13.4%)
Undergraduate and above	12 (3.1%)
The highest level of education of the participant’s mother	
Primary school and below	156 (40.2%)
Secondary or technical secondary school	184 (47.4%)
Higher vocational School	36 (9.3%)
Undergraduate and above	12 (3.1%)

### 3.2. Measures

#### 3.2.1. Learning engagement scale

Learning engagement was assessed using the Chinese version of the Utrecht Work Engagement Scale for Students (Gan et al., 2007). The scale has previously been used to research Chinese educational contexts (e.g., Wu et al., 2020; Shang et al., 2022; Wang et al., 2022). It consists of 17 items and 3 dimensions: vigor, dedication, and absorption. The items were rated on a 5-point Likert scale (1 = never; 5 = every day), with higher scores reflecting stronger learning engagement. The Cronbach’s alpha coefficient for this study was 0.94.

#### 3.2.2. Career calling scale

The two-item Brief Calling Scale was adapted from Dik et al. (2012) and has briefly been used to measure career calling in non-Chinese (Duffy and Autin, 2013; Douglass and Duffy, 2015; Duffy et al., 2017) and Chinese contexts (Zhang et al., 2015; Cai et al., 2022). The two items are “I have a sense of calling to nursing” and “I am very aware of the calling that nursing should have.” The items were rated on a five-point Likert scale (1 = strongly disagree; 5 = strongly agree), with higher scores reflecting higher levels of career calling. The Cronbach’s alpha coefficient in this study was 0.70.

#### 3.2.3. Career adaptability scale

Career adaptability was assessed using the Career Adaptability Scale (Hou et al., 2012; Porfeli and Savickas, 2012). This scale has been widely used in previous studies with both non-Chinese (İspir et al., 2019; Safavi and Bouzari, 2019; Boo et al., 2021) and Chinese participants (Hou et al., 2019; Xu et al., 2020; Boo et al., 2021) to assess career adaptability. The 24-question scale consists of 4 dimensions: career concern, career control, career curiosity, and career confidence, with 6 questions in each dimension. The items were rated on a five-point Likert scale (1 = strongly disagree; 5 = strongly agree), with higher scores reflecting higher levels of career adaptability. The Cronbach’s alpha coefficient for this study was 0.96.

#### 3.2.4. Career commitment scale

The Career Commitment Scale was adapted from Blau (1989) and was developed through previous studies with non-Chinese (Ahmed, 2017; Afsar et al., 2019) and Chinese participants (Huang et al., 2019). The scale consists of seven items rated on a five-point Likert scale (1 = strongly disagree; 5 = strongly agree), with higher scores reflecting higher levels of career commitment. The Cronbach’s alpha coefficient of the scale in this study was 0.94.

### 3.3. Data analysis

Preliminary analysis was conducted using SPSS (v. 26.0) to identify participants’ characteristics, measure the reliability of each scale, and analyze the correlations between the four variables of career calling, learning engagement, career adaptability, and career commitment. To test the fit of the model, a validated factor analysis (CFA) was conducted using the Amos software (v. 25.0) measurements of *x*^2^/df, RMSEA, CFI, and TLI. According to Hu and Bentler (1999), a model is considered acceptable if the values returned are as follows: *x*^2^/df ≤ 5, RMSEA <0.08, and CFI/TLI > 0.9. A model is considered a good fit if the value of *x*^2^/df ≤ 5, RMSEA < 0.055, and CFI and TLI > 0.95. Finally, the mediation test was performed using the SPSS PROCESS macro.

## 4. Results

### 4.1. Common method bias test

In this study, Harman’s single-factor test was used to test for common method bias (Eby and Dobbins, 1997). The exploratory factor analysis of the 50 questions showed that the first factor explained 39.08% of the variance, below the 40% threshold, thereby ruling out any significant common method bias.

### 4.2. Confirmatory factor analysis

To examine the discriminant validity between the four variables of career calling, learning engagement, career commitment, and career adaptability, a validated factor analysis was conducted. As shown in Table 2, compared with the three-factor, two-factor, and single-factor models, the four-factor model provided the best model fit (*χ*^2^ = 197.466, df = 98, *x*^2^/df = 2.015, RMSEA = 0.043, CFI = 0.923, and TLI = 0.906). This confirmed the variables possessed good discriminant validity.

**Table 2:** Confirmatory factor analysis results (*N* = 388).

Model	*x* ^2^	df	*x*^2^/df	RMSEA	CFI	TLI
Four factors	197.466	98	2.015	0.043	0.923	0.906
Three factors	439.864	101	4.355	0.187	0.739	0.69
Two factors	603.057	103	5.855	0.225	0.615	0.552
Single factors	619.484	104	5.957	0.227	0.603	0.542

Four factors = career calling, learning engagement, career commitment, and career adaptability; three factors: career calling, learning engagement, and career commitment + career adaptability; two factors: career calling, learning engagement + career commitment + career adaptability; single factors: career calling + learning engagement + career commitment + career adaptability.

### 4.3. Descriptive analysis

Descriptive statistics and correlation analyses were conducted for each variable (see Table 3). Career calling was significantly and positively correlated with learning engagement (*r* = 0.324, *p* < 0.01) and career adaptability (*r* = 0.295, *p* < 0.01), and strongly and positively correlated with career commitment (*r* = 0.538, *p* < 0.01). Career adaptability (*r* = 0.521, *p* < 0.01) and career commitment (*r* = 0.470, *p* < 0.01) showed a significant positive correlation with learning engagement.

**Table 3:** Means, standard deviations, and correlations.

Items	*M*	SD	1	2	3	4
1. Career calling	3.495	0.734	-			
2. Career adaptability	3.384	0.660	0.295^**^	-		
3. Career commitment	3.298	0.786	0.538^**^	0.482^**^	-	
4. Learning engagement	3.351	0.615	0.342^**^	0.521^**^	0.470^**^	-

*N* = 388, ^∗^*p* < 0.05, ^∗∗^*p* < 0.01, and ^∗∗∗^*p* < 0.001.

### 4.4. Hypothesis testing

To further determine the relationship between the variables, we conducted regression analyses (see Table 4). Model 6 controlled for demographic variables and showed a significant positive correlation between career calling and engagement in learning (*β* = 0.424, *p* < 0.01), thereby supporting hypothesis 1.

**Table 4:** Regression analysis of parallel mediator model variables.

Variables	Career adaptability		Career commitment		Learning engagement			
	Model 1	Model 2	Model 3	Model 4	Model 5	Model 6	Model 7	Model 8
Categories of high school	−0.055	0.081	−0.317	−0.001	−0.357	0.149	0.09	0.121
College application	0.005	0.066	−0.108	−0.146	0.357	0.113	0.089	0.129
Career calling		0.341^**^		0.483^***^		0.424^**^		0.176
Career adaptability							0.364^***^	0.347^**^
Career commitment							0.336^**^	0.267^*^
*R* ^2^	0.003	0.927	0.119	0.308	0.004	0.149	0.349	0.369
*F*	0.140	3.336^*^	6.368	13.783^***^	0.187	5.433^**^	12.330^***^	10.623^***^

^*^p < 0.05, ^**^p < 0.01, ^***^p < 0.001.

To verify the parallel mediating effects of career adaptability and career commitment on the relationship between career calling and learning engagement, we applied MacKinnon’s multiple mediating effects test MacKinnon (2000). This has three steps: first, to test whether the effect of the independent variable on the dependent variable is significant; second, to test the significance of the independent variable’s effects on multiple mediating variables and whether multiple mediating variables significantly affect the dependent variable; third, to test whether the effect of the mediating variables on the dependent variable is significant by including multiple mediating variables in the regression equation of the dependent on the independent variable.

The first result of these tests suggested that career calling had a positive and significant effect on learning engagement. Secondly, as Models 2 and 4 demonstrated, the mediating variables (career adaptability and career commitment) were both positively influenced by the respondents’ sense of purpose (*β* = 0.341, *p* < 0.01; *β* = 0.483, *p* < 0.001). Thirdly, as shown in Model 7, career adaptability and career commitment both had a significant positive mediating effect on learning engagement (*β* = 0.346, *p* < 0.001; *β* = 0.336, *p* < 0.01). Finally, Model 8 demonstrated that the regression coefficient of career calling on learning engagement decreased from 0.424 to 0.176 and became non-significant after adding both career adaptability and career commitment while the coefficients of career adaptability (*β* = 0.347, *p* < 0.01) and career commitment (*β* = 0.267, *p* < 0.05) on learning engagement were both significant. This result indicates that both career adaptability and career commitment mediated the relationship between career calling and learning engagement, thereby confirming hypotheses 2 and 3.

To further test the significance of the mediating effect of career adaptability and career commitment on the link between career calling and engagement in learning, a bias correction test for the mediating effect was conducted following the steps described by Preacher and Hayes (2008). The results of the parallel mediating effect model test are shown in Table 5. First, the direct effect of career calling on learning engagement was 0.148 at a 95% confidence interval [−0.027, 0.323], an insignificant direct effect. Second, the indirect effect of career calling on learning engagement through career adaptability was 0.099, 95% CI [0.028, 0.232], establishing a significant mediating effect for career adaptability in the relationship. Finally, the indirect effect of career calling on learning engagement through career commitment was 0.108, with a 95% CI [0.013, 0.189]. This result shows that career commitment significantly mediated the relationship between career calling and learning engagement (see Table 5).

**Table 5:** Bootstrap analyses of significance of mediation.

Model pathways	Effect	Bootstrap SE	95% Confidence interval		Percentage
			Boot LLCI	Boot ULCI	
Indirect effect	0.207	0.071	0.078	0.356	58.31%
Direct effect	0.148	0.088	−0.027	0.323	
Career adaptability	0.099	0.041	0.028	0.232	27.89%
Career commitment	0.108	0.039	0.013	0.189	30.42%
Career adaptability—Career commitment	−0.008	0.070	−0.145	−0.128	

## 5. Discussion

This study aimed to build understanding of the relationship between career calling, career commitment, career adaptability, and learning engagement. Specifically, we hypothesized that career calling would directly or indirectly predict the learning engagement of vocational nursing students in the Chinese context through vocational commitment and career adaptability. Our results confirmed this hypothesis, which is consistent with previous studies on the relationship between career calling, learning outcomes and personal wellbeing (Duffy et al., 2011a, 2014; Duffy and Dik, 2013; Shang et al., 2022). The findings also suggested that career calling is an important psychological resource for strengthening students’ motivation and engagement in learning. For these students, career calling is manifested as a transcendent summoning or guiding force that aligns their career with a broader sense of life’s meaning and purpose, and an intention to help others or advance the greater good (Li et al., 2021). Learners with stronger career callings will be more actively involved in considering and planning their own careers. As a result, they will invest more time and energy in their studies to build a solid foundation for their long-term professional development.

To further investigate the relationship between career calling and learning engagement, two mediating variables—career adaptability and career commitment—were used to build a parallel mediating effect model. The variables mediated the relationship between career calling and learning engagement fully and in parallel, thereby verifying hypotheses 2 and 3. This result strongly suggests that nursing students with a strong career calling were more engaged in their studies due to their commitment and adaptability to their careers. In other words, a high level of career calling may promote career commitment and career adaptability among nursing students, in turn boosting their academic engagement. This supports Douglass and Duffy’s (2015) previous uncovering of a positive relationship between career calling and career adaptability (Douglass and Duffy, 2015), as well as Ke et al.’s (2020) finding that career commitment mediates the relationship between career calling and student learning behavior. The present study therefore confirmed potential roles for career commitment and career adaptability in building the psychological resources that help students deal with challenging learning tasks. One possible explanation is that if learners are more focused on their future occupation, while maintaining strong senses of responsibility for future occupational development, keen curiosity about future occupational opportunities, and the adaptability to solve job-related problems, they will remain positive when facing challenges to learning.

## 6. Theoretical implications

Many studies of educational quality have explored how to enhance learning engagement levels. However, rather than examining the effects of teaching styles, strategies, content, and teacher-student interaction on engagement, our study focused on the intrinsic expectations and career aspirations of individuals. We hypothesized that career calling, commitment, and adaptability might positively impact engagement by buffering individuals against the effects of stress caused by challenging learning circumstances. Our findings emphasized the importance of career calling, career commitment, and career adaptability for learning engagement and offered theoretical insights into how to enhance students’ academic performance.

## 7. Practical implications

This study also guides careers education by highlighting that career calling, adaptability, and commitment can all enhance students’ learning engagement and academic performance. Specifically, teachers should optimize course content and teaching activities to raise awareness of the importance and potential benefits of a career-focused mindset *via* activities such as role-play. Such modifications would build a sense of professional purpose among nursing students, prompting them to invest more time and energy in their learning. Schools and teachers should also attend more closely to career planning and guidance so students are fully aware of the opportunities available to them. Goals can be assigned for students to work towards. Finally, career calling, career adaptability, and career commitment could also be used to diagnose why some students struggle to complete their programs and provide solutions to such educational issues.

## 8. Conclusion

In this study, we investigated the associations between career calling, learning engagement, career adaptability, and career commitment by analyzing self-reported data from 388 nursing students. We also examined the mediating effects of career adaptability and career commitment on the relationship between career calling and learning engagement. Using a multiple mediation model, we demonstrated that career vocation positively affected learning engagement and that this relationship was mediated by career adaptability and career commitment. The study contributes to theoretical knowledge of career calling and learning engagement while also informing educators, policymakers, and administrators about the motivational aspects of nurse education.

## 9. Research limitations and future research

Nonetheless, the study also contains several limitations. First, the cross-sectional design made it impossible to establish any longitudinal causal relationships between career calling, career adaptability, career commitment, and learning engagement. We therefore plan to study the pathways between these variables longitudinally to fully understand the impact of career calling on nursing students’ learning engagement over time as a means of improving the quality of nurse education programs. A second limitation is that this study has considered the mediating role of career adaptability as a whole but its four separate indicators (concern, control, curiosity, and confidence) may play different roles in this relationship—an issue that requires further exploration. Third, the study findings are based on the responses of higher vocational nursing students in a province in central China and their generalizability to other regions of the country or to nursing students at the undergraduate level and above will require further research. Fourth, the study used a self-administered questionnaire, which may lead to common method bias. Accordingly, subsequent studies should gather data through multiple channels to enhance the validity of the findings.

## Data availability statement

The raw data supporting the conclusions of this article will be made available by the authors, without undue reservation.

## Ethics statement

The studies involving human participants were reviewed and approved by Zhengzhou Railway Vocational & Technical College. The patients/participants provided their written informed consent to participate in this study.

## Author contributions

All authors listed have made a substantial, direct, and intellectual contribution to the work, and approved it for publication.

## Funding

This study was supported by the Higher Education Teaching Reform Research and Practice Project of Henan.

## Conflict of interest

The authors declare that the research was conducted in the absence of any commercial or financial relationships that could be construed as a potential conflict of interest.

## Publisher’s note

All claims expressed in this article are solely those of the authors and do not necessarily represent those of their affiliated organizations, or those of the publisher, the editors and the reviewers. Any product that may be evaluated in this article, or claim that may be made by its manufacturer, is not guaranteed or endorsed by the publisher.
