# Supplementary material for: The impact of career calling on higher vocational nursing students’ learning engagement: The mediating roles of career adaptability and career commitment
Source: Front Psychol. 2023 Mar 22;14:1111842. doi: 10.3389/fpsyg.2023.1111842 (PMC10075228; doi:10.3389/fpsyg.2023.1111842)
Supplement: Supplementary file 1 [file Data_Sheet_1.docx]

# Appendix

**The questionnaire of nursing students’ career calling and learning engagement**

**Instructions: Thank you for taking part in this survey. The aim of this anonymous survey is to gain an understanding of nursing students' career calling and learning engagement, as well as the main influencing factors. Your responses will only be used for statistical analysis and will remain confidential. It is essential that you answer truthfully and accurately in order to ensure the accuracy of our study.**

**Part One: The basic situation**

1. Your gender:

A. Male B. Female

2. Your Grade:

A. Freshman B. Sophomore C. Junior

3. Where are you come from?

A. Rural towns

B. Small and medium-sized cities

C. Big cities

4. Which categories of high school do you attend

A. Secondary Vocational and Technical College

B. Ordinary High School

C. Key High School

5. Is college application what you want it to be?

A. Yes B. No

6. What is the highest level of education your father has achieved?

A. Primary school and below

B. Secondary or technical secondary school

C. Higher Vocational School

D. undergraduate and above

7. What is the highest level of education your mother has achieved?

A. Primary school and below

B. Secondary or technical secondary school

C. Higher Vocational School

D. undergraduate and above

**Part Two: Calling Career Scale**

**Instruction: Read each item and compare it to your feelings; then select the level that corresponds to your level of conformity from the checkboxes below.**

| Question | 1. complete disagreement | 2. disagree | 3. uncertainty | 4. agree | 5. complete agreement | |
| --- | --- | --- | --- | --- | --- | --- |
| 1. I have a calling to nursing. | 1 | 2 | 3 | 4 | 5 |  |
| 2. I have a good understanding of my calling as it applies to my career | 1 | 2 | 3 | 4 | 5 |  |

**Part Three: learning engagement**

**Instruction: Read each item and compare it to your feelings; then select the level that corresponds to your level of conformity from the checkboxes below.**

| Question | 1. completely disagreement | 2. disagree | 3. uncertainty | 4. agree | 5. completely agreement |
| --- | --- | --- | --- | --- | --- |
| 1. As soon as I wake up in the morning, I am happy to go and study | 1 | 2 | 3 | 4 | 5 |
| 2. I feel energised when I study | 1 | 2 | 3 | 4 | 5 |
| 3. Even when learning doesn’t go well, I don't get discouraged and I persevere | 1 | 2 | 3 | 4 | 5 |
| 4. I can study for a long time without taking a break | 1 | 2 | 3 | 4 | 5 |
| 5. I can recover quickly from mental fatigue when studying | 1 | 2 | 3 | 4 | 5 |
| 6. I feel strong and motivated when I am studying | 1 | 2 | 3 | 4 | 5 |
| 7. I find learning challenging | 1 | 2 | 3 | 4 | 5 |
| 8. Learning inspires me | 1 | 2 | 3 | 4 | 5 |
| 9. I am passionate about learning | 1 | 2 | 3 | 4 | 5 |
| 10. I am proud of my learning | 1 | 2 | 3 | 4 | 5 |
| 11. I find that my learning is purposeful and meaningful | 1 | 2 | 3 | 4 | 5 |
| 12. When I learn, I forget everything around me | 1 | 2 | 3 | 4 | 5 |
| 13. When I study, I feel that time flies | 1 | 2 | 3 | 4 | 5 |
| 14. When I study, all I can think about is my studies | 1 | 2 | 3 | 4 | 5 |
| 15. I find it hard to let go of my studies | 1 | 2 | 3 | 4 | 5 |
| 16. I am immersed in my studies | 1 | 2 | 3 | 4 | 5 |
| 17. I feel happy when I am fully engaged in my studies | 1 | 2 | 3 | 4 | 5 |

**Part Four: career adaptability scale**

**Instruction: Read each item and compare it to your feelings; then select the level that corresponds to your level of conformity from the checkboxes below.**

| Question | 1. completely disagreement | | 2. disagree | 3. certainty | 4. agree | | 5. completely agreement |
| --- | --- | --- | --- | --- | --- | --- | --- |
| 1. Thinking about what my future will be like | | 1 | 2 | 3 | 4 | 5 | |
| 2. Realizing that today's choices shape my future | | 1 | 2 | 3 | 4 | 5 | |
| 3. Preparing for the future | | 1 | 2 | 3 | 4 | 5 | |
| 4. Becoming aware of the educational and career choices that I must make | | 1 | 2 | 3 | 4 | 5 | |
| 5. Planning how to achieve my goals | | 1 | 2 | 3 | 4 | 5 | |
| 6. Concerned about my career | | 1 | 2 | 3 | 4 | 5 | |
| 7.Keeping upbeat | | 1 | 2 | 3 | 4 | 5 | |
| 8. Making decisions by myself | | 1 | 2 | 3 | 4 | 5 | |
| 9. Take responsibility for my actions | | 1 | 2 | 3 | 4 | 5 | |
| 10. Sticking up for my beliefs | | 1 | 2 | 3 | 4 | 5 | |
| 11. Counting on myself | | 1 | 2 | 3 | 4 | 5 | |
| 12. Doing what's right for me | | 1 | 2 | 3 | 4 | 5 | |
| 13. Explore my surroundings | | 1 | 2 | 3 | 4 | 5 | |
| 14. Look for opportunities to grow as a person | | 1 | 2 | 3 | 4 | 5 | |
| 15. Investigating possible opportunities before making a choice | | 1 | 2 | 3 | 4 | 5 | |
| 16. Observe different ways of doing things | | 1 | 2 | 3 | 4 | 5 | |
| 17. Probing deeply into questions I have | | 1 | 2 | 3 | 4 | 5 | |
| 18. Being curious about new opportunities | | 1 | 2 | 3 | 4 | 5 | |
| 19. Performing tasks efficiently | | 1 | 2 | 3 | 4 | 5 | |
| 20. Taking care to do things well | | 1 | 2 | 3 | 4 | 5 | |
| 21. Learn new skills | | 1 | 2 | 3 | 4 | 5 | |
| 22. Working up to my ability | | 1 | 2 | 3 | 4 | 5 | |
| 23. Overcomes obstacles | | 1 | 2 | 3 | 4 | 5 | |
| 24. Solve problems | | 1 | 2 | 3 | 4 | 5 | |

**Part Five: Career Commitment Scale**

**Instruction: Read each item and compare it to your feelings; then select the level that corresponds to your level of conformity from the checkboxes below.**

| Question | 1. completely disagreement | 2. disagree | | 3. uncertainty | | 4. agree | | 5. completely agreement | |  | |
| --- | --- | --- | --- | --- | --- | --- | --- | --- | --- | --- | --- |
| 1.I think nursing is a very desirable career and I would definitely like to work in nursing in the future | 1 | | 2 | 3 | 4 | | 5 | |  | |  |
| 2.I would not give up nursing even if a new career opportunity with better pay came along | 1 | | 2 | 3 | 4 | | 5 | |  | |  |
| 3.If I had to do it all over again, I would still like to work in nursing | 1 | | 2 | 3 | 4 | | 5 | |  | |  |
| 4.I would love to develop myself in the nursing profession | 1 | | 2 | 3 | 4 | | 5 | |  | |  |
| 5.I would still like to work in nursing even if I had enough money without having to work | 1 | | 2 | 3 | 4 | | 5 | |  | |  |
| 6.I am glad that I chose nursing as a career | 1 | | 2 | 3 | 4 | | 5 | |  | |  |
| 7.I think nursing is a very desirable career and worthy of a lifelong commitment | 1 | | 2 | 3 | 4 | | 5 | |  | |  |
